# Power Failure of Mitochondria and Oxidative Stress in Neurodegeneration and Its Computational Models

**DOI:** 10.3390/antiox10020229

**Published:** 2021-02-03

**Authors:** JunHyuk Woo, Hyesun Cho, YunHee Seol, Soon Ho Kim, Chanhyeok Park, Ali Yousefian-Jazi, Seung Jae Hyeon, Junghee Lee, Hoon Ryu

**Affiliations:** 1 Brain Science Institute, Korea Institute of Science and Technology, Seoul 02792, Korea; wjh601@kist.re.kr (J.W.); eileen316@kist.re.kr (H.C.); yseol@kist.re.kr (Y.S.); soonhokim@kist.re.kr (S.H.K.); chpark@kist.re.kr (C.P.); yousefian@kist.re.kr (A.Y.-J.); t15321@kist.re.kr (S.J.H.); 2 Department of Physics and Astronomy and Center for Theoretical Physics, Seoul National University, Seoul 08826, Korea; 3 Department of Neurology, Boston University Alzheimer’s Disease Center, Boston University School of Medicine, Boston, MA 02118, USA; junghee@bu.edu; 4 VA Boston Healthcare System, Boston, MA 02130, USA

**Keywords:** mitochondria, oxidative stress, antioxidants, Alzheimer’s disease, amyotrophic lateral sclerosis, Huntington’s disease, Parkinson’s disease, computational modeling

## Abstract

The brain needs more energy than other organs in the body. Mitochondria are the generator of vital power in the living organism. Not only do mitochondria sense signals from the outside of a cell, but they also orchestrate the cascade of subcellular events by supplying adenosine-5′-triphosphate (ATP), the biochemical energy. It is known that impaired mitochondrial function and oxidative stress contribute or lead to neuronal damage and degeneration of the brain. This mini-review focuses on addressing how mitochondrial dysfunction and oxidative stress are associated with the pathogenesis of neurodegenerative disorders including Alzheimer’s disease, amyotrophic lateral sclerosis, Huntington’s disease, and Parkinson’s disease. In addition, we discuss state-of-the-art computational models of mitochondrial functions in relation to oxidative stress and neurodegeneration. Together, a better understanding of brain disease-specific mitochondrial dysfunction and oxidative stress can pave the way to developing antioxidant therapeutic strategies to ameliorate neuronal activity and prevent neurodegeneration.

## 1. Introduction

The brain uses around 20% of energy in the whole adult human body [1,2,3]. Because brain cells consume high levels of energy, they require a powerful generator of energy. The mitochondrion is a subcellular organelle and a power engine that produces adenosine-5′-triphosphate (ATP), the biochemical “energy currency” in cells. Glucose yields pyruvate (pyruvic acid) through the process of glycolysis. Pyruvate is transported to the mitochondria and generates hydrogen ions via a pathway of oxidative phosphorylation. Pyruvate dehydrogenase oxidizes pyruvate to form acetyl coenzyme A that is oxidized in the tricarboxylic acid cycle. The coenzymes released in these mentioned pathways, the reduced form of nicotinamide adenine dinucleotide (NADH) and flavin adenine dinucleotide (FADH2) are oxidized in the respiratory chain, resulting in the generation of a proton gradient between the inner and outer membrane of the mitochondria. An increase in electrochemical potential gradient by protons drives a membrane potential (negative inside) and a pH gradient (basic inside) that synthesizes ATP through F_0_F_1_-ATP synthase.

Mitochondria produce 80% or more of the reactive oxygen species (ROS) in brain cells. In general, oxidative stress is an indispensable process and an essential cellular event that occurs naturally [4]. Herein, an outstanding question arises: how does it become detrimental and toxic so as to damage macromolecules and many different types of cells in the brain [5,6]? A plausible answer may be that oxidative stress becomes toxic when it continuously accelerates and reaches beyond a threshold level. Produced by a leakage of electrons at the level of four complexes (I–IV), ROS, such as superoxide anion radical (O_2_^−^), hydrogen peroxide (H_2_O_2_), and hydroxyl radical (^•^OH) results in DNA oxidation, lipid peroxidation, and protein oxidation.

Superoxide dismutase 1 (SOD1) catalyzes the partitioning of two superoxide anion (2O_2_^•^^−^) and converts it into hydrogen peroxide and oxygen. Then, hydrogen peroxide is processed by catalase to produce two water molecules and oxygen. Aging and environmental stressors attenuate brain cell function to neutralize ROS and in consequence, oxidative stress disrupts cellular homeostasis and triggers the progression of neurodegeneration. The vicious cycle of oxidative stress is known to be the most common pathogenesis in neurodegenerative disorders including Alzheimer’s disease (AD), amyotrophic lateral sclerosis (ALS), Huntington’s disease (HD), Parkinson’s disease (PD), and others [3,7,8,9]. In this review, we discuss which molecules and stressors affect mitochondrial function and how oxidative stress is linked to the pathophysiology and neurodegeneration of AD, ALS, HD, and PD, respectively [3,7,8,9,10]. Furthermore, we present computational modeling studies on mitochondria-driven metabolic processes and oxidative stress.

## 2. Brain Disorder and Mitochondrial Dysfunction

### 2.1. Alzheimer’s Disease (AD)

Alzheimer’s disease (AD) is a common neurodegenerative disease characterized by impaired cognition and neuronal loss in aged people. Principal agents of AD pathology are attributed to the intracellular aggregation of hyperphosphorylated tau and the abnormal deposition of amyloid beta (Aβ), which may be oligomeric (oAβ) or aggregated into senile plaques. Recently, significant evidence of mitochondria as the direct targets for Aβ mediated neuronal-toxicity has been produced. Marked by a decrease in ATP production and an increase in oxidative stress elements such as ROS, mitochondrial dysfunction contributes to AD pathogenesis [10]. One area of interest is transcription factor p53, a vital component of aerobic respiration. Under normal circumstances, p53 trans-activates cytochrome oxidase 2 (SCOS), a subunit of cytochrome c oxidase (COX) or complex IV from the electron transport chain (ETC). However, it is well known that under pathogenic conditions, oAβ upregulates p53, leads to increased expression of SCOS, and increases ROS production [11]. Although additional p53 is recruited from the nucleus to the mitochondria to ensure cell survival, p53 dependent anti-oxidative capacities are greatly suppressed in AD, in which p53 will begin to exhibit prooxidative activities [12,13]. 

In AD mitochondria, antioxidant checkpoint mechanisms are significantly reduced. When returning to mitochondrial homeostasis, NAD-dependent deacetylase sirtuin-3 (SIRT3), a class III histone deacetylase localized in the inner mitochondrial membrane and matrix, decreases abundant p-53. Furthermore, when SIRT3 protein levels are reduced, Lee et al. discovered increased mito-p53 occupancy [14]. Recent RNA expression chip datasets have confirmed downregulated SIRT3 mRNA levels compared to controls in post-mortem AD brains and in 3xTG AD mice expressing microtubule associated protein tau (MAPT), amyloid-beta precursor protein (APP), and presenilin-1(PSEN1) transgenes [15]. Notably, AD patients exhibited decreased expression of mitochondrial DNA (mtDNA) encoded subunits of complex I (ND2 and ND4), nuclear DNA encoded subunits of complex I (NDUFA2), and nuclear DNA encoded subunits of complex IV (NDUFA4) [14,16,17]. Given that the mitochondrial respiratory complexes remain critical for normal ATP production, any subunit functional defects can reduce respiratory activity and increase ROS production. 

Concurrently, mitochondria-specific Aβ and p53 accumulation revealed mitochondrial oxidative events as an element not only sufficient to induce pathological conditions but also result in neuronal death [12,18,19,20]. For instance, under oxidative stress, ROS damages nuclear DNA, triggering assembly of the DNA damage response network [20]. Especially when DNA double-stranded breaks (DBS) occur, p53 is phosphorylated by ATM serine/threonine kinase (ATM) [21]. However, depending on the extent of DNA damage, p53 will either drive gene expression towards the activation of cell cycle checkpoints or cell death checkpoints. It has been reported that p53 transactivates critical determinants of mitochondrial apoptosis such as Bax, Bak, Nova, and Puma while repressing those that are antiapoptotic such as Bcl-2, Bcl-X, and Mcl-1 [22]. p53 may also directly activate Bax and inhibit Bcl-2/-X in the mitochondria, driving the assembly of Bax/Bak lipid pores, which alters mitochondrial membrane potential (ΔΨ) and leads to mitochondrial apoptosis [23]. 

In addition, p53 also engages with the mitochondrial permeability transition pore via direct interaction with cyclophilin D (CypD) [24]. CypD regulates the opening of mitochondrial permeability transition pore by phosphorylating serine residue S191 [25]. Indeed, SIRT3 which deacetylates CypD is downregulated in AD [26]. Furthermore, oAβ-CypD complexes were found in AD brains and transgenic AD mice [27,28]. Mitochondrial permeability transition pore activation results in decreased ΔΨ. An influx of cytosolic molecules, induces mitochondrial swelling and eventual rupture of the outer membrane, ending in mitochondrial necrosis [29]. 

**Figure 1:**
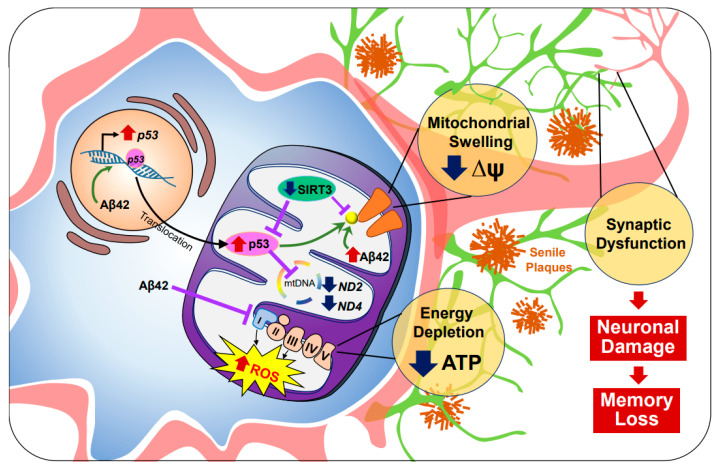
Mitochondrial dysfunction in Alzheimer’s disease. Oligomeric amyloid beta (oAβ) accumulates intracellularly and triggers mitochondrial dysfunction in Alzheimer’s disease (AD). In the nucleus, oAβ upregulates the expression of p53. While stress signals are present, p53 is translocated to the mitochondria. Sirtuin-3 (SIRT3), which inhibits p53 as well as cyclophilin D (CypD) activity (yellow) functions as a reactive oxygen species (ROS) modulator, but is downregulated, tipping the balance of respiration in favor of ROS production. While oAβ inhibits electron transport chain activity, p53 also represses the production of ND2 and ND4, subunits of complex I, contributing towards overall energy depletion. Subsequently, low ATP levels disturb downstream processes, such as axonal transportation, resulting in synaptic dysfunction. Ultimately, over accumulated p53 and oAβ activates CypD, which prolongs mitochondrial permeability transition pore permeability, triggering cell death.

Overall, a loss of ΔΨ often activates both apoptotic/necrotic pathways as well as mitophagy in the same cell. Damaged mitochondria are selectively degraded by autophagy in an effort to prevent cell death. However, under pathogenic conditions, mitophagy or protective mechanisms are significantly compromised [30,31]. Simultaneously, impairment of mitochondrial trafficking has been reported in AD hippocampal neurons [32,33]. Mitochondria deficiency at the synapse, decreases available synaptic ATP essential for neurotransmission [18,34]. In combination, ranging from apoptosis/necrosis to mitophagy, mitochondrial dynamics remain as a complex interdependent system that is significantly disturbed, leading to neuronal death in the AD brain (Figure 1). 

There still lacks a clear mechanism of the various elements that influence changes from normal conditions to abnormal conditions in AD mitochondria. Higher oAβ concentrations were correlated with lower SIRT3 levels in a dose-dependent manner, as shown on neuronal cells [35]. oAβ can also bind directly to and suppress complex I, accelerating Aβ toxicity [36,37,38]. This supports the notion that mitochondria are a site of a deleterious positive feed-back loop of toxic oAβ, exacerbating AD pathogenesis [39]. Moreover, studies have shown that Aβ jeopardizes neuronal viability via mitochondria in a non-cell autonomous manner. In microglia, the transcriptional upregulation of nucleotide-binding oligomerization domain (NOD)-like receptor protein (NLRP3) inflammasome components combined with the generation of Aβ induced ROS, triggers the assembly of the NLRP3 inflammasome. NLRP3 inflammasome activation is then amplified by cross seeding Aβ, which ultimately results in pyroptotic cell death (pyroptosis). Microglia releases free functional Aβ, consequently injuring neighboring neurons. In total, oAβ directly interacts with multiple downstream levels of mitochondria dysfunction in a cell specific manner. Thus, ameliorating suppressed anti-oxidative capacities in relation to Aβ toxicity in AD may be a potential therapeutic target warranting further investigation. 

### 2.2. Amyotrophic Lateral Sclerosis (ALS)

**Figure 2:**
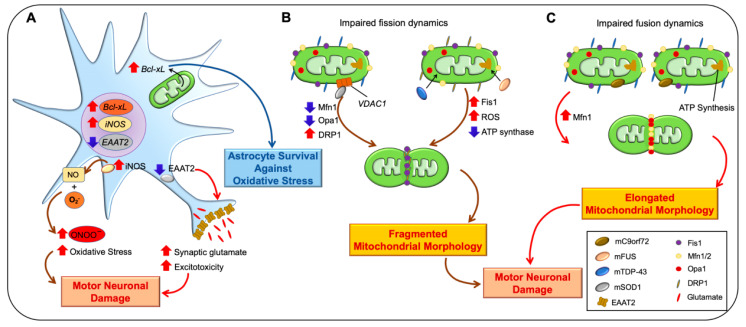
Mitochondrial dysfunction in amyotrophic lateral sclerosis (ALS). (**A**) B-cell lymphoma-extra large (Bcl-xL) gene expression is increased in astrocytes in response to cellular stress. The increase of Bcl-xL prevents oxidative damage in astrocytes under ALS conditions. Furthermore, inducible nitric oxide synthase (iNOS) expression in reactive astrocytes facilitates an interaction of superoxide (O_2_^−^) with nitric oxide (NO) that forms peroxynitrite (ONOO^-^). Moreover, reduction of excitatory amino acid transporter 2 (EAAT2) levels inhibits glutamate uptake in the synaptic cleft, elevates the synaptic glutamate, and triggers motor neuronal damage and cell death. (**B**) Mutated FUS and mutated TAR DNA-binding protein 43 (mTDP-43) are localized to mitochondria, increase mitochondrial fission 1 protein (Fis1) and ROS levels, and subsequently decrease ATP synthesis activity. In addition, mutated superoxide dismutase 1 (SOD1), FUS, and TDP-43 impair mitochondrial fission dynamics and lead to mitochondrial fragmentation and apoptosis. (**C**) Mutated C9orf72 increase mitofusion-1 (Mfn1) and abnormal mitochondrial fusion.

Amyotrophic lateral sclerosis (ALS) is an adult-onset fatal neurodegenerative disorder that affects the motor neurons located in the spinal cord, brain stem, and motor cortex. Progressive motor neuron loss leads to muscle weakness, then muscle atrophy, spasticity, eventual paralysis and finally death 3 to 5 years after diagnosis by denervation of the respiratory muscles [40]. ALS-associated mitochondrial dysfunction occurs at different levels, including ROS production, defective oxidative phosphorylation, oxidative stress, and defective mitochondrial dynamics [8]. Superoxide (O_2_^−^) and nitric oxide (NO), two key pathogenic components produced by the electron transport system (ETS), leads to oxidative damage such as DNA oxidation, protein oxidation, and lipid peroxidation [9,41]. High levels of O_2_^−^ can interact with NO to form peroxynitrite (ONOO^-^) which may strengthen oxidative stress and trigger motor neuron damage [42,43,44]. Furthermore, our group has previously demonstrated that mitochondrial B-cell lymphoma-extra large (Bcl-xL) gene expression is increased in astrocytes in response to cellular stress. An increase in mitochondrial Bcl-xL prevents oxidative damage in astrocytes under ALS conditions in mutant (G93A SOD1) transgenic mice (Figure 2A) [45,46]. In the ALS model, excitatory amino acid transporter 2 (EAAT2) expression is reduced in reactive astrocytes, lowering glutamate uptake in the synaptic cleft. Subsequently, the high level of extracellular synaptic glutamate leads to excitotoxicity in the spinal cord of ALS patients [46,47].

Mitochondria are highly dynamic organelles whose structural alterations—mitochondrial fission and fusion—may contribute to the etiology of ALS (Figure 2B,C) [48]. Mitochondrial metabolites are shared and ΔΨ is dissipated during the fusion process. Motility acceleration and damaged part sequestration occurred during mitochondrial fission before mitochondrial disposal [49]. Mitochondrial fusion is governed by mitofusin1 (Mfn1), mitofusin2 (Mfn2), and optic atrophy protein1 (OPA1) [50]. Whereas dynamin-related protein1 (Drp1) and fission1 (Fis1) promote mitochondrial fission (Figure 2B,C) [51]. Disruption of mitochondrial cristae has been exhibited in C9orf72-related, mutated SOD1-, TARDBP-, and FUS-associated ALS cases [52,53,54,55]. Interestingly, either cellular stress or pathogenic-variant conditions facilitate trans-localization of FUS and TDP-43 to the mitochondria. They increase Fis1 levels that cause mitochondrial fragmentation, loss of membrane potential, and consequently an increase in ROS production and defective mitochondrial axonal transport (Figure 2B) [56]. Furthermore, FUS interacts with mitochondrial ATP synthase and reduces ATP production in mitochondria that cause a loss of mitochondrial cristae, and mitochondrial fragmentation [57]. Liu et al. demonstrated that the reduction of mitochondrial fusion proteins (Mfn1 and Opa1) and the steady-state activity of Drp1 and Fis1 are important for mitochondrial fission by altering the balance of mitochondrial morphology in ALS mutant (G93A SOD1) transgenic mice (Figure 2B) [58]. Notably, a dynamic change of fission and fusion is also shown in mC9orf72 fibroblasts with elevated MFN1 levels, resulting in an abnormal enlargement of mitochondrial morphology (Figure 2C) [53]. Together, given the contribution of mitochondrial dysfunction to the pathogenesis of ALS, identifying how mitochondrial dysfunctions are relevant to disease onset and progression will elucidate important targets for the development of neuroprotective therapies in ALS. 

### 2.3. Huntington’s Disease (HD)

Huntington’s disease (HD) is an autosomal dominant neurodegenerative disease caused by a polymorphic CAG repeat extension in exon 1 of the huntingtin gene, which encodes for mutant Huntingtin (mHTT) protein [59]. Although the exact function of mHTT is unknown, it interacts with other proteins involved in mitochondrial function, energy metabolism, calcium handling, and transcriptional regulation. Under HD conditions, mutant huntingtin (mHTT) protein becomes aggregated and generates nuclear inclusion bodies. They also make cytoplasmic aggregates that directly interact with mitochondrial elements [60]. It has previously been found that complex I activity was decreased in the platelets of HD patients [61,62]. In addition, a significant decrease in complexes III and IV activity was observed in the caudate and putamen of HD patients, which contributes to a reduction in ΔΨ [63,64,65]. Succinate dehydrogenase (SDH), a main component of Krebs cycle and complex II, was also markedly reduced in the caudate nucleus of HD brains [66]. SDH affects mitochondrial function, catalyzing the oxidation of succinate to fumarate in the Krebs cycle, reducing ubiquinone to ubiquinol. 3-nitropropionic acid (3-NP) and malonate act as an irreversible and reversible inhibitor of SDH, respectively [67,68,69]. In chemical models of HD, malonate and 3-NP decreases Krebs cycle, complex II, and mitochondrial respiratory chain complex activity. It is believed that malonate-induced energy deficits cause a partial membrane depolarization, resulting in excitatory toxicity by removing the voltage-dependent magnesium block of NMDA receptor-gated calcium channels [70,71,72]. 

**Figure 3:**
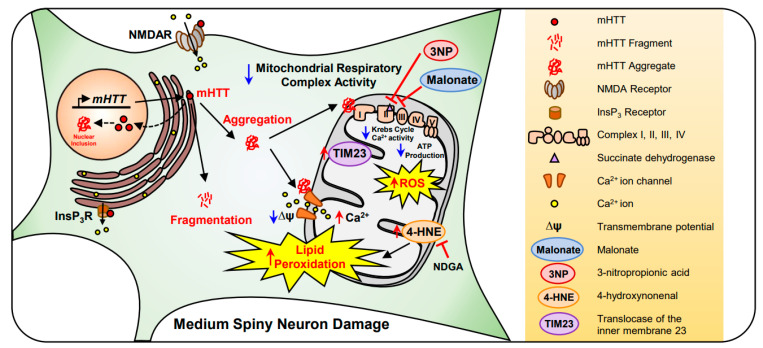
Mitochondrial dysfunction and lipid peroxidation are found in the medium spiny neuron (MSN) of HD. mutant Huntingtin (*mHTT)* gene expresses mHTT protein in the MSN. mHTT inclusions are increased in the nucleus and cytosol. In the cytoplasm, both increased mHTT protein fragments and aggregates triggers neuronal damage. The aggregated form of mHTT decreases Krebs cycle and Ca^2+^ activity, resulting in a decrease in complex I–IV or mitochondrial respiratory complex activity. mHTT aggregation also decreases mitochondrial membrane potential (ΔΨ) and increases reactive oxygen species (ROS) production. As Ca^2+^ permeability increases, the mitochondrial membrane is hyperpolarized, triggering cell loss. In chemical models of Huntington’s disease (HD), malonate and 3-nitropropionic acid (3NP) inhibits succinate dehydrogenase (SDH) at complex II and, eventually, reduces ATP production via complex V. Lastly, the level of 4-hydroxynonenal (4-HNE), a neuronal lipid peroxidative damage marker, is elevated in HD and nordihydroguaiaretic acid (NDGA) reduces 4-HNE levels and prevents mitochondrial damage in the MSN.

In HD, mHTT inhibits protein importation in the mitochondria of isolated brains by overexpressing translocase from the inner membrane 23 (TIM23), leading to neuronal death in HD [73]. In addition, mHTT decreases ∆ψm stability, triggering impaired mitochondrial Ca^2+^ homeostasis, and abnormal mitochondrial trafficking [74]. It has been shown that mHTT enhances N-methyl-D-aspartate receptor (NMDAR), voltage-gated Ca^2+^ channel, and inositol trisphosphate receptor (InsP3R) activity in a HD transgenic (YAC128) mouse model [75,76,77,78]. Mitochondrial ATP production is significantly reduced in the muscles of both symptomatic HD patients and presymptomatic HD gene carriers [79]. Moreover, the generation of ROS highly impacts oxidative phosphorylation and lipid peroxidation in the mitochondria of HD patients. Lipid peroxidation and mitochondrial dysfunction are correlated in the striatum of HD patients and HD animal models [80,81]. Lipid peroxidation is a metabolic process in which ROS leads to the oxidative degradation of lipids. 4-hydroxynonenal (4-HNE), a lipid peroxidation product, is significantly elevated in the caudate and putamen of the human HD brain. Nordihydroguaiaretic acid (NDGA), a flavonoid and an antioxidant, decreased the levels of 4-HNE and mHTT and restored ΔΨ in R6/2 HD mice. Together, mHTT directly affects mitochondrial dysfunction and leads to neuronal death in HD (Figure 3). Therapeutic modulations of mitochondrial function and avoidance of oxidative stress are beneficial to HD [81]. In this regard, the development of antioxidant drugs for targeting mitochondria may expedite the treatment of HD. 

### 2.4. Parkinson’s Disease (PD)

Parkinson’s disease (PD) is a progressive neurodegenerative disorder that is characterized by unusual motor, non-motor symptoms, and the loss of dopaminergic neurons [82]. The relation between mitochondrial damage and PD was first reported in the substantia nigra of PD patients in 1989 [83,84,85]. In the substantia nigra of the patient brain there is approximately a 35% deficiency in mitochondrial respiratory complex I [86]. This is supported by the administration of 1-methyl-4-phenyl-1,2,3,6-tetrahydropyridine (MPTP), a prodrug which inhibits complex I and causes oxidative stress damage in dopaminergic neurons [87]. 

In the dopaminergic neuron, gene mutations linked to mitochondrial dysfunction have been identified and their pathogenetic mechanisms have been studied in PD. For example, mutations of phosphatase and tensin homolog (PTEN)-induced kinase (PINK1), Parkin, and DJ-1 are closely associated with mitochondrial dysfunction and abnormal autophagy in PD patients [88,89]. Mutation of Omi/HtrA2 gene (G399S) results in a severe parkinsonian phenotype [90,91]. Moreover, glucocerebrosidase (GBA) L444P mutation causes a mitochondrial defect in primary neurons, affecting PD [92]. Together, it is apparent that gene mutations and oxidative stress are closely associated with dopaminergic neuronal damage and pathogenesis of PD. 

**Figure 4:**
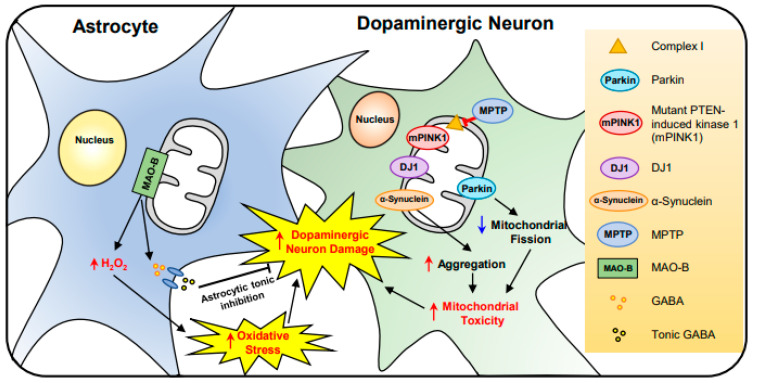
The effect of mitochondrial dysfunction on astrocytes and neurons in Parkinson’s disease (PD). In the dopaminergic neuron, 1-methyl-4-phenyl-1,2,3,6-tetrahydropyridine (MPTP) causes a parkinsonian syndrome characterized by a loss of dopamine-producing neurons. Autosomal recessive mutations in Parkin, Phosphatase and tensin homolog (PTEN)-induced kinase 1 (PINK1), and DJ-1 increases mitochondrial toxicity. Thus, elevating α-Synuclein aggregation and mitochondrial degradation. On the other hand, in reactive astrocytes, monoamine oxidase B (MAO-B) produces GABA, which is released in a tonic manner, affecting neuronal degeneration. As a result, MAO-B activity also induces oxidative stress via H_2_O_2_ that ultimately triggers the damage of dopaminergic neurons.

Astrocytes provide structural and metabolic support to neurons, regulate blood flow, water transport, and synaptic transmission within the brain [93]. They also produce various neurotrophic molecules including glial-derived neurotrophic factor (GDNF) for the development and survival of dopaminergic neurons [94,95]. In contrast, reactive astrocytes trigger the disruption of the blood–brain barrier and affect the substantia nigra of PD patients [96]. Interestingly, our group recently found that reactive astrocytes elevate the activity of flavin-dependent monoamine oxidase B (MAO-B) and produce H_2_O_2_ in the substantia nigra of PD patients. This study shows a novel pathological mechanism of PD that reactive astrocytes release gamma-aminobutyric acid (GABA), an inhibitory neurotransmitter, and the astrocytic tonic inhibition causes dopaminergic neuronal damage (Figure 4) [97]. This finding suggests that reactive astrocytes are involved in non-cell autonomous pathway of dopaminergic neuronal damage, and therapeutic regulation of this pathway may be applicable for improving neuropathological and behavioral abnormality in PD. 

## 3. Computational Modeling of Mitochondria in Brain Disorders

Computational modeling has been used to explore the role of mitochondrial dysfunction in neurodegenerative diseases. The nature of computational approaches allows zooming in the details of metabolic pathways within a mitochondrion or zooming out to describe the population dynamics of mitochondria. Computational models and simulations are especially useful for the understanding of the organelle’s function in a quantitative manner. Furthermore, they can provide information on the onset and progression of the diseases by bridging the gap between experimental studies.

### 3.1. Computational Modeling of Mitochondria

The main focus of the computational approaches at a single mitochondrion scale has been on the energy-producing machinery in a network of dynamic intracellular enzymatic reactions [98,99,100,101,102,103,104,105,106]. Traditionally, the computational modeling of this scale has been either thermodynamic, kinetic, or stoichiometric approaches [107]. Thermodynamic models, based on fundamental principles of thermodynamics, are simple approaches for the understanding of complex molecular pathways and for testing various hypotheses, but they lack mechanistic details [108,109,110,111,112]. To examine the biochemical details, one may use stoichiometric models [113,114,115], which, however, cannot offer information on transient behavior or regulatory interactions of metabolic pathways. Kinetic models can be used to describe transient behavior and regulatory interactions to unveil kinetics and mechanistic details of the pathways. However, when quantitative kinetic data from experiments is lacking, the model is only useful to understand the phenomenological behaviors of the pathways [103,116]. 

**Figure 5:**
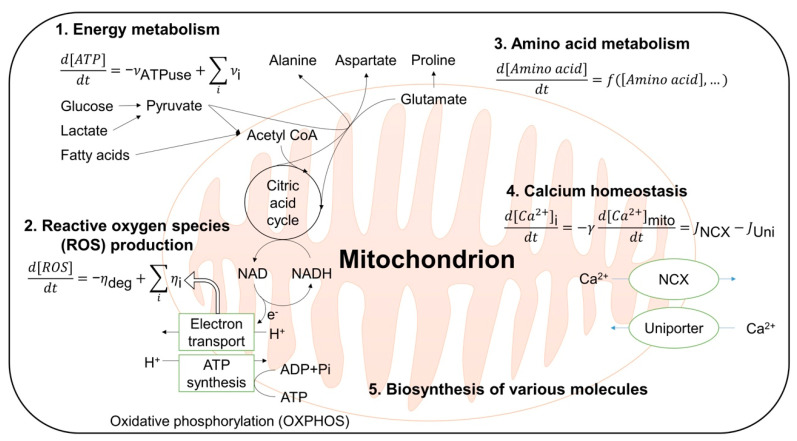
Computational modeling in a single mitochondrion scale. The major roles of a mitochondrion are illustrated schematically and example equations are displayed. [X] represents the concentration of the molecule X. *ν*_ATPuse_ is the rate of ATP usage, *ν*_i_ the ATP production rate of *i*-th pathway, *η*_deg_ the rate of ROS degradation, *η*_i_ the ROS production rate of *i*-th pathway, *γ* the volume ratio of the mitochondrion to the cytoplasm, and *J* the flux of calcium ions through the channel in the subscript. *f* can be any function, usually polynomial function, of amino acid and other intermediates concentrations. NCX refers to the sodium–calcium exchanger.

The modeling and simulations can examine not only the details of modular metabolic pathways of a mitochondrion, but can also be used to describe interactions of these pathways [117,118]. The major pathways (Figure 5) include energy metabolism [97,98,99,100,101,102,103,104,105], ROS production [117,118,119,120,121,122], amino acid metabolism [123], calcium homeostasis [124,125], and biosynthesis of various molecules [126] (Figure 5). Dysfunctions of these pathways are critical for the development of neurodegenerative diseases directly or indirectly via ROS-related pathways [19,127].

**Figure 6:**
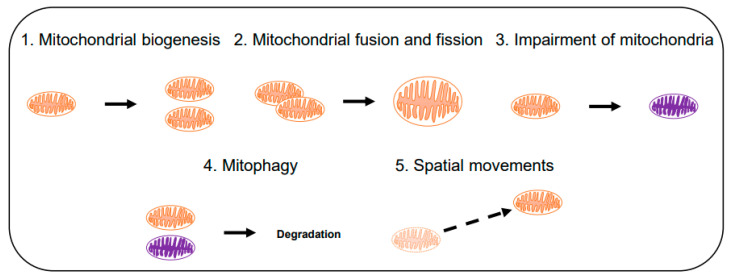
Computational modeling of mitochondria at a population scale. Major pathways for mitochondrial population dynamics are illustrated. Orange and purple colored mitochondria represent normal and impaired mitochondria, respectively.

Figure 6 illustrates the representative computational modeling approaches for the population dynamics of mitochondria. Mitochondrial biogenesis (pathway 1 in Figure 6) and mitophagy (autophagic degradation of mitochondria; pathway 4) regulates the number of mitochondria in a cell. Mitophagy [128] occurs selectively to reduce the number of impaired mitochondria that are generated via pathway 3 [129]. Fusion (pathway 2, forward) helps mitochondria to survive against external stress and fission (pathway 2, backward) creates new mitochondria while keeping the quality of the population [130]. The spatial movement of mitochondria (pathway 5) helps mitochondria to enter other pathways like fusion and mitophagy. The mitochondrial population can also respond to stresses, such as hypoxia and calcium flux, by changing its spatial distribution [131]. Computational studies can explain how these pathways are related to the maintenance of mitochondrial functions against internal and external stressors [132,133,134,135,136,137,138,139,140].

The ATP synthase, also called the F_1_F_0_-ATPase, plays a critical role in mitochondrial function [141]. It is found deregulated in AD [142,143,144], which may lead to neuronal dysfunction [145,146], and it is linked to numerous other neurological diseases [147,148,149]. Modeling of the kinetics as well as the molecular dynamics may play an increasingly important role in understanding ATP synthase dysfunction in neurodegeneration.

**Figure 7:**
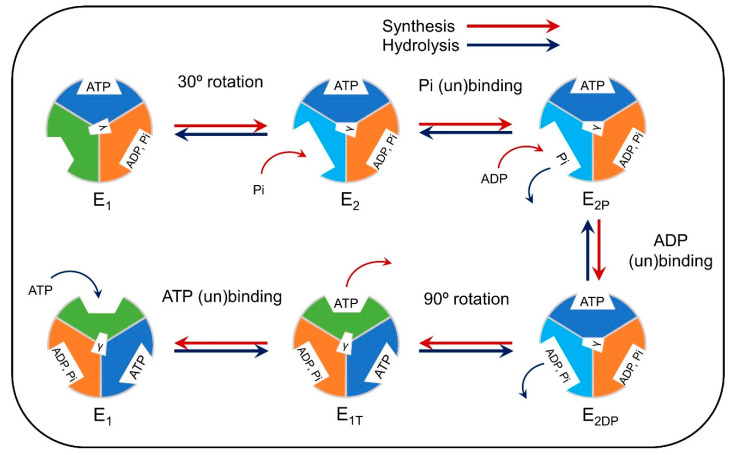
Schematic of the reaction cycle of the F_1_ subcomplex for ATP synthesis and hydrolysis. The sequence of ATP synthesis is shown by red arrows, and ATP hydrolysis is indicated by blue arrows. After one cycle, the ATPase returns to an equivalent configuration but is rotated by 120°. The color and shape of each catalytic binding site indicate their structure with dark blue, orange, green, and light blue corresponding to structures β_TP_, β_DP_, β_E_, and β_HC_, respectively [14]. The sequence of ATP synthesis is shown by red arrows, and ATP hydrolysis is indicated by blue arrows. The orientation of the γ subunit is depicted at the center of the synthase.

The ATP synthase uses the proton-motive force across the inner membrane to synthesize ATP from ADP and phosphate. It can also perform the opposite reaction, which results in ATP hydrolysis. Early kinetic models include Pietrobon and Caplan’s six-state model [150], which was incorporated in models of mitochondrial metabolism [102,103]. Gao et al. [151] incorporated structural details of the complex (Figure 7) [152,153,154,155]. Figure 7 shows a rotational cycle corresponding to the synthesis or hydrolysis of one ATP, and the three β subunits of the ATP synthase are drawn for each step of the cycle. The color and shape of the subunits correspond to their conformational states, which mediate substrate binding. The conformational changes of the β subunits are induced by the rotation of the γ subunit (whose orientation is depicted in the schematic), the rotation of which is driven by the proton-motive force [151]. 

Several partial high-resolution crystal structures of the mitochondrial F_1_F_0_-ATPase have been found for yeast and bovine heart [154,155,156,157,158], making possible molecular dynamics (MD) simulations that can help elucidate the workings of the ATPase in atomistic detail. MD simulations of the F_1_ subcomplex have provided insight into the transfer of mechanical energy by applying rotations on the γ subunit [159,160]. Simulations of the c_10_ ring of the F_0_ subcomplex provided insight into the proton binding mechanism [161,162]. Several simulations of the F_0_ motor investigated the ATP synthase’s relation to MPTP. An MD study of the c_10_ ring concluded that the lumen of the ring cannot be occupied by water and so it cannot be the MPTP [163]. However, a Ca^2+^ binding site on the β subunit of mitochondrial ATPase has been identified which could induce a conformational change transmitted to the lateral stalk and cause the permeability transition [164]. Recent advances in membrane molecular simulations using multi-scale methods have allowed simulations of a whole mitochondrial membrane [165], with promising future applications.

### 3.2. Application of Mitochondrial Modeling in Neurodegenerative Diseases

**Figure 8:**
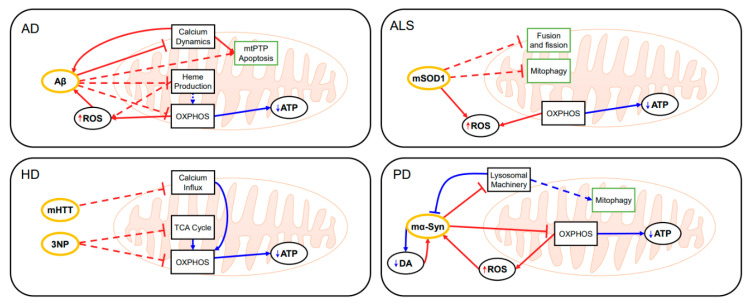
Mitochondrial mechanisms of major neurodegenerative diseases including Alzheimer’s disease (AD), Parkinson’s disease (PD), Huntington’s disease (HD), and amyotrophic lateral sclerosis (ALS). Ovals and rectangles are molecules and pathways, respectively. Sharp/blunt arrows represent positive/negative interactions. Interactions that are enhanced in the disease condition are colored in blue, while the diminished are colored in red. Pathways in a single mitochondrion scale are colored in black and pathways in a population scale are colored in green. Interactions that have not been computationally modeled are represented in dashed lines. Aβ, amyloid-beta; ROS, reactive oxygen species; OXPHOS, oxidative phosphorylation; mtPTP, mitochondrial permeability transition pore; mα-Syn, mutant α-synuclein; DA, dopamine; TCA, tricarboxylic acid; mHTT, mutant huntingtin; 3NP, 3-nitropropionic acid; mSOD1, mutant superoxide dismutase 1.

Computational modeling has been used to understand the roles of mitochondria in neurodegenerative diseases, including AD and PD (Figure 8) [49,166,167,168,169]. Among the computational studies of AD, the model of Hao et al. considered the effect of ROS, yet detailed mitochondrial ROS production mechanisms were omitted [170]. Their model of AD included neurons, astrocytes, microglia and peripheral macrophages, as well as amyloid beta aggregation and hyperphosphorylated tau proteins. The model was used to simulate the effect of drugs, which requires many resources to test without computational model, and suggested the efficient therapy for slowing the progression of AD. Ranjan et al. developed a computational model for the interaction among amyloid-beta, calcium signaling, and mitochondrial permeability transition pore (mtPTP) related cell apoptosis in AD [171]. The model was used to simulate calcium dynamics in the presence of amyloid beta and showed the increased concentration of intracellular calcium ions and dysregulation of Ca^2+^ channel receptors on the endoplasmic reticulum.

Raichur et al. developed in 2006, one of the earliest computational models of PD focusing on the aggregation and clearance of mα-Syn as influenced by the ROS generation [166]. They showed that increased oxidative stress can generate a result similar to 1-methyl-4-phenyl-1,2,3,6-tetrahydropyridine (MPTP), which is known to induce PD. Cloutier et al. also developed the computational models of PD, of which the relation between mitochondrial ROS generation and α-synuclein aggregation was investigated [167,169]. They further extracted an equivalent two-state feedback motif from the model and proposed a principle for PD pathogenesis in the form of the two-state transition between “healthy” and “disease” states.

Accumulating evidence indicates that mitochondria may play an important role in HD and ALS [48,172] in addition to AD and PD as discussed above. Among them, autophagy is one of the most important and versatile mechanisms to understand the onset and progression of neurodegenerative diseases. Therefore, the computational modeling of autophagy [173,174,175,176,177] should be further developed to include detailed mitochondrial mechanisms. Further studies for computational modeling of the interaction between Aβ/mHTT/mSOD1/mα-Syn and mitochondria would offer a useful framework to understand the complex dynamics of the brain disease. 

## 4. Conclusions

The energy transfer from non-living things to living organisms occurs through catabolism and anabolism of organic carbon molecules. Though the generation of ROS is an inevitable cellular event that happens during the catabolism in the mitochondria, apparently they become harmful factors to the brain, ultimately exacerbating neurodegeneration. By understanding how ROS-mediated oxidative stresses damages the brain, it will be possible to develop therapeutic strategies and antioxidant drugs to prevent neurodegeneration. To do so, future research will need to address several challenges as identified below.

First, it will be important to define the brain cell-type specific mechanisms of oxidative stress. Considering the fact that the brain consists of many different cell types such as neurons (excitatory neurons versus inhibitory neurons) and non-neuronal cells (astrocyte, microglia, oligodendrocyte, endothelial cells, pericytes, and ependymal cells), it is proposed that oxidative stress-responses are various and signal transduction pathways are differentially regulated in a brain cell type- and region-specific manner. In this paradigm, oxidative stress can be toxic or become pro-death signals to neurons while it can be pro-survival signals to non-neuronal cells. Indeed, gliogenesis, the generation of new astrocytes and microglia, is commonly found under oxidative stress-induced pathological conditions [178]. Accordingly, future study is required to determine what exact molecular pathways are triggered by oxidative stress and how these pathways influence the fate of neuron and glia selectively and respectively. It is also important to identify the threshold level of oxidative stress that ultimately causes autonomous cell death versus non-cell autonomous cell death in the brain. A growing body of evidence has proven that ROS from reactive astrocytes and microglia are detrimental to pyramidal neurons in the hippocampus, motor neurons in the spinal cords, medium spiny neurons in the striatum, and dopaminergic neurons in the substantia nigra [179].

Second, identification of *bona fide* early oxidative stress markers in the brain will expedite the diagnosis and treatment of neurodegenerative processes. Not only is the identification of specific molecules and signaling cascades crucial for understanding the pathogenesis of neurological disorders, but it is also pivotal in discovering treatments for oxidative damages in the brain. Recent findings show that mitochondria-dependent cellular events are emerging as potential therapeutic targets. The coping of acute oxidative stresses at the earlier time point may protect brain cells more effectively than that at a time point that is too late to balance the above threshold of oxidative damages, which could then become irreversible and degenerative. Perhaps constitutive generation of ROS can transform reactive glia to become foes of neurons and thereby propagate neuronal cytotoxicity throughout the brain [179]. In this paradigm, any therapeutic approaches to ameliorate oxidative stresses in neurodegenerative disorders should consider the time window and cell-type/brain-region specificity of drug effects. 

Lastly, as we summarized in Section 3, useful computational models of mitochondria have been made over a wide range of scales. Some of these models have yet to be used to observe the mitochondria-dependent mechanisms of neurodegenerative diseases. The advance of computational modeling will expedite the understanding of the dynamic function of mitochondria and oxidative stress in neurodegenerative diseases. Together, we hope our review can accelerate modeling studies for the comprehensive understanding of neurodegenerative diseases, as well as contribute towards the development mitochondria-specific antioxidant therapeutics.
